# Beyond the Lungs: A Rare Encounter With Tuberculous Colitis in the Colon

**DOI:** 10.7759/cureus.100569

**Published:** 2026-01-01

**Authors:** Marie Jo Geha, Hassan Buhulaigah, Srijana Bajracharya, Antonio Topacio, Kevin Chan, Sanja Krajisnik, Olaniyi Fadeyi

**Affiliations:** 1 Internal Medicine, West Anaheim Medical Center, Anaheim, USA; 2 Pathology, Garden Grove Hospital and Medical Center, Garden Grove, USA

**Keywords:** anti-tuberculosis treatment, diagnostic challenges, extrapulmonary tuberculosis (eptb), inflammatory bowel disease, tuberculosis colitis

## Abstract

Intestinal tuberculosis is a very rare manifestation of bacterial tuberculosis. Affected patients often present with nonspecific symptoms which could mimic Crohn’s disease, intestinal cancer, or amebiasis. Consequently, the presentation of intestinal tuberculosis remains a diagnostic challenge. Here, we report the case of a 27-year-old female with a past medical history of external hemorrhoids who presents to the hospital with complaints of generalized weakness, dizziness, watery diarrhea, significant weight loss, and near syncope. CT abdomen and pelvis with oral contrast revealed colonic wall thickening suggestive of colitis as well as bibasilar reticulonodular opacities and left basilar cavitary lesions concerning for infection with suspicion for mycobacterial organisms. AFB smear was positive for TB. Colonoscopy revealed some ulcers, which were biopsied. Results of the biopsy revealed diffuse granulomatous colitis in the cecum and active granulomatous colitis in the ascending and descending colon. The patient was eventually diagnosed with ITB colitis. Her symptoms improved with antituberculosis medications.

## Introduction

Tuberculosis (TB) is a bacterial infection caused by Mycobacterium tuberculosis complex (MBTC). Some members of the MBTC include *Mycobacterium tuberculosis*, *Mycobacterium africanum*, *Mycobacterium bovis*, and *Mycobacterium canetti*. According to the World Health Organization (WHO), TB is one of the leading causes of infectious disease-related deaths worldwide [1]. It primarily affects the pulmonary system, but extrapulmonary manifestations have been seen in several cases. While pulmonary TB accounts for 80%-85% of all TB cases, extrapulmonary TB has been documented in about 20% of all affected patients [2]. Intestinal tuberculosis (ITB) is a very rare form of extrapulmonary TB seen in only 1-3% of all cases [3]. While ITB is occasionally diagnosed in immunocompetent individuals, the occurrence of ITB is mostly seen in TB endemic areas and among immunocompromised patients [3]. Symptoms of ITB are nonspecific, and unless a high index of suspicion is maintained, the diagnosis can be missed or delayed, resulting in increased morbidity and mortality [4]. Affected patients typically present with nonspecific symptoms (abdominal pain, weight loss, fever, change in bowel habits) which could mimic Crohn’s disease, amoebiasis, or colon cancer [5]. Accurate diagnosis is very crucial to facilitate an appropriate treatment delivery. Wrong or delayed diagnosis could result in complications such as stricture, blockage, colonic perforation, and bleeding [6]. We hereby report the case of a 27-year-old immunocompetent female who presented with generalized weakness, weight loss, watery diarrhea, and dizziness. She was eventually diagnosed with intestinal tuberculosis.

## Case presentation

**Figure 1:**
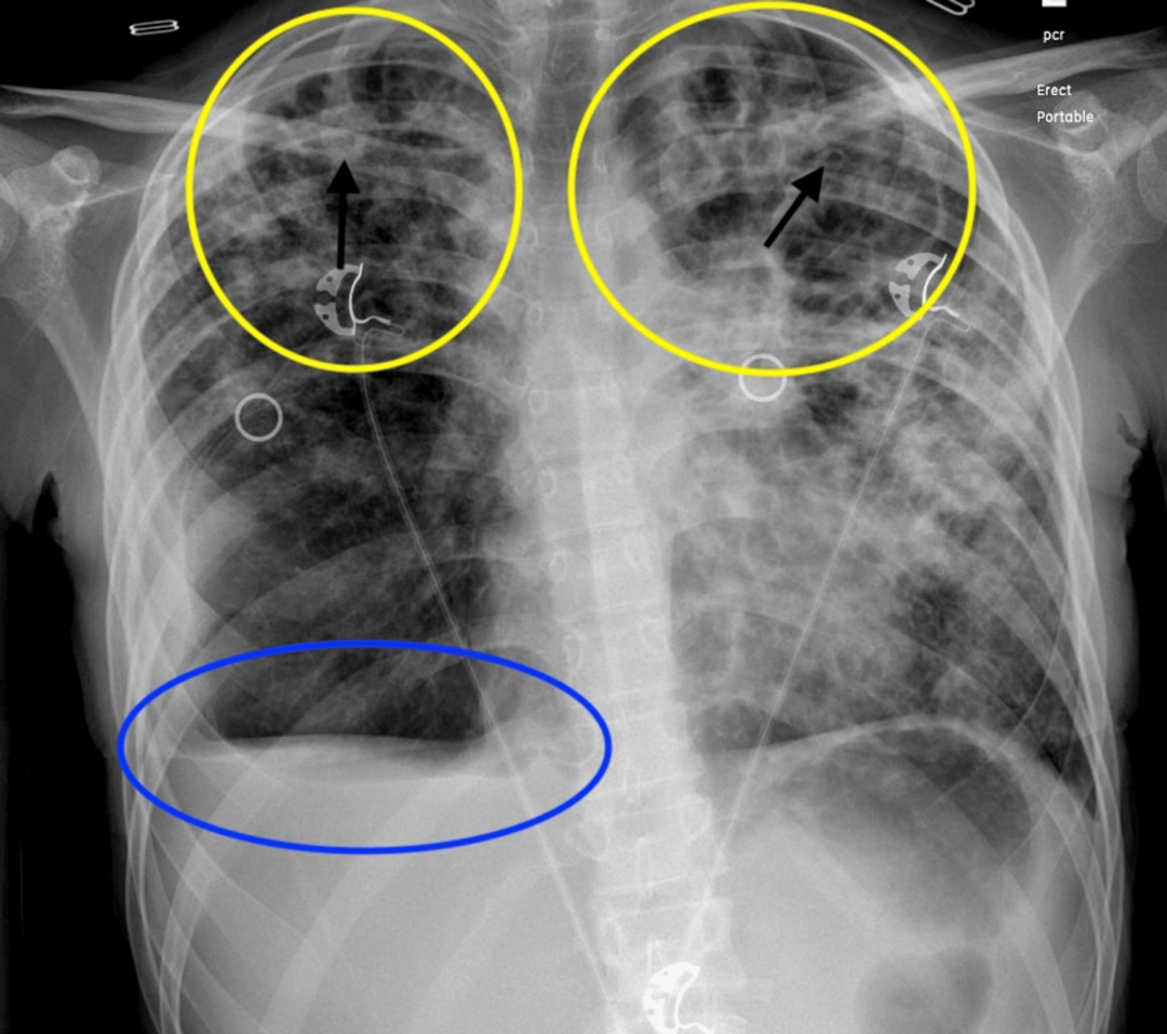
Chest X-ray demonstrating bilateral upper lobe ground glass opacities (yellow circles) with interlobular and peribronchial thickening (black arrows) alongside pleural effusion with blunting of the costophrenic angle (blue oval).

A 27-year-old female patient with a past medical history of external hemorrhoids presented to the hospital with complaints of generalized weakness, dizziness, watery diarrhea, significant weight loss, and near syncope. Her symptoms have been persistent for a few weeks, but worsened some days prior to presentation. Initial assessment showed BMI at 11.94 kg/m². On assessment, the patient was afebrile, tachycardic, borderline hypotensive, and mildly hypoxic. Initial labs revealed leukocytosis, microcytic anemia, and thrombocytosis (WBC: 17.5x10^9/L; reference range 4.80-10.80x10^9/L, hemoglobin: 9.7 g/dL; reference range 12-15.5 g/dL, mean corpuscular value: 68.6 fL; reference range 81.6-98.3 fL, and platelet: 802x10^9/L; reference range 150-450x10^9/L). Chest X-ray revealed bilateral upper lobe dominant ground glass densities along with interlobular and peribronchial thickening suggestive of multifocal pneumonia as well as a small right-sided pleural effusion (Figure 1).

**Figure 2:**
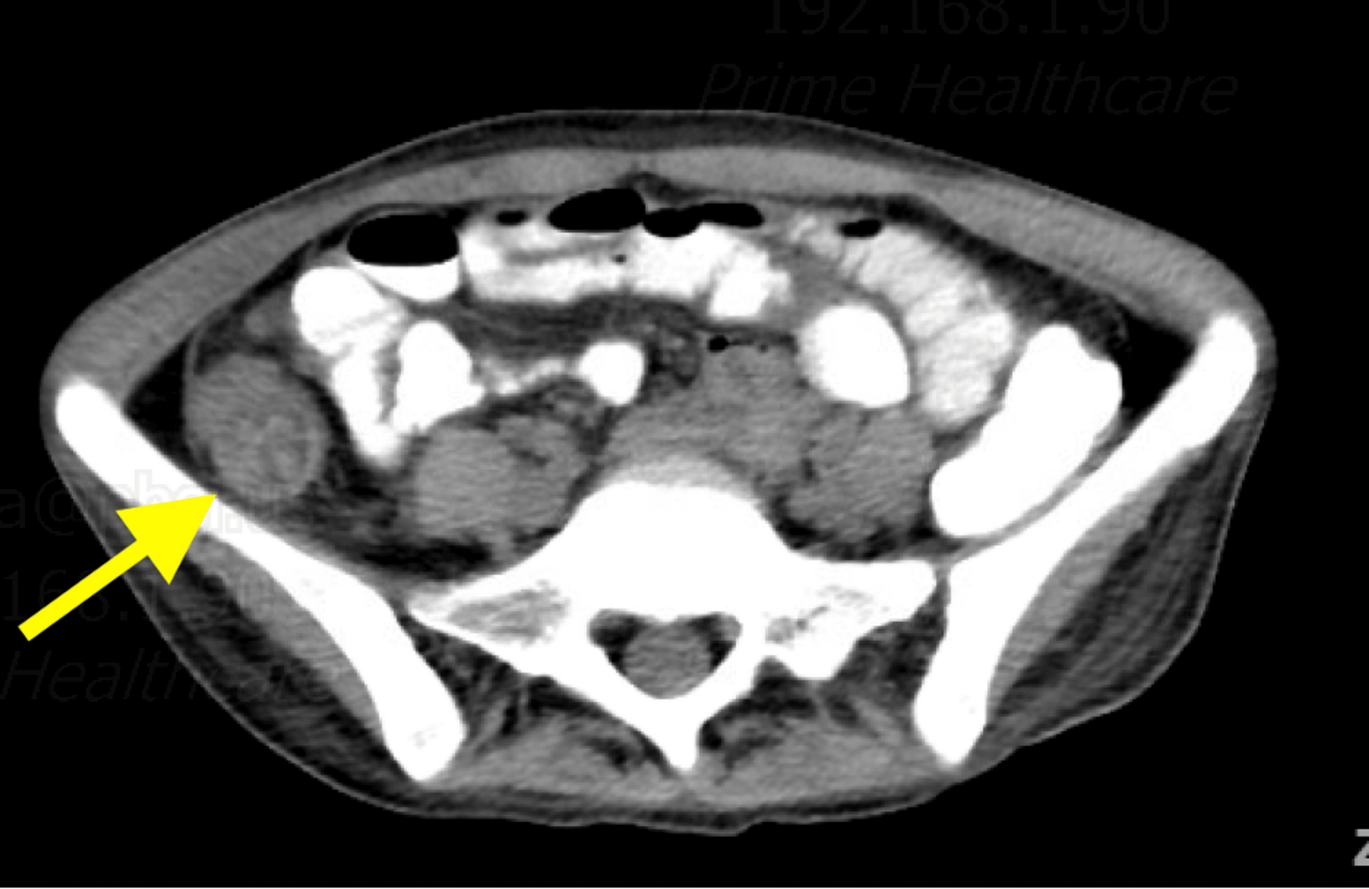
CT of the abdomen and pelvis revealing wall thickening of the ascending colon (yellow arrow).

**Figure 3:**
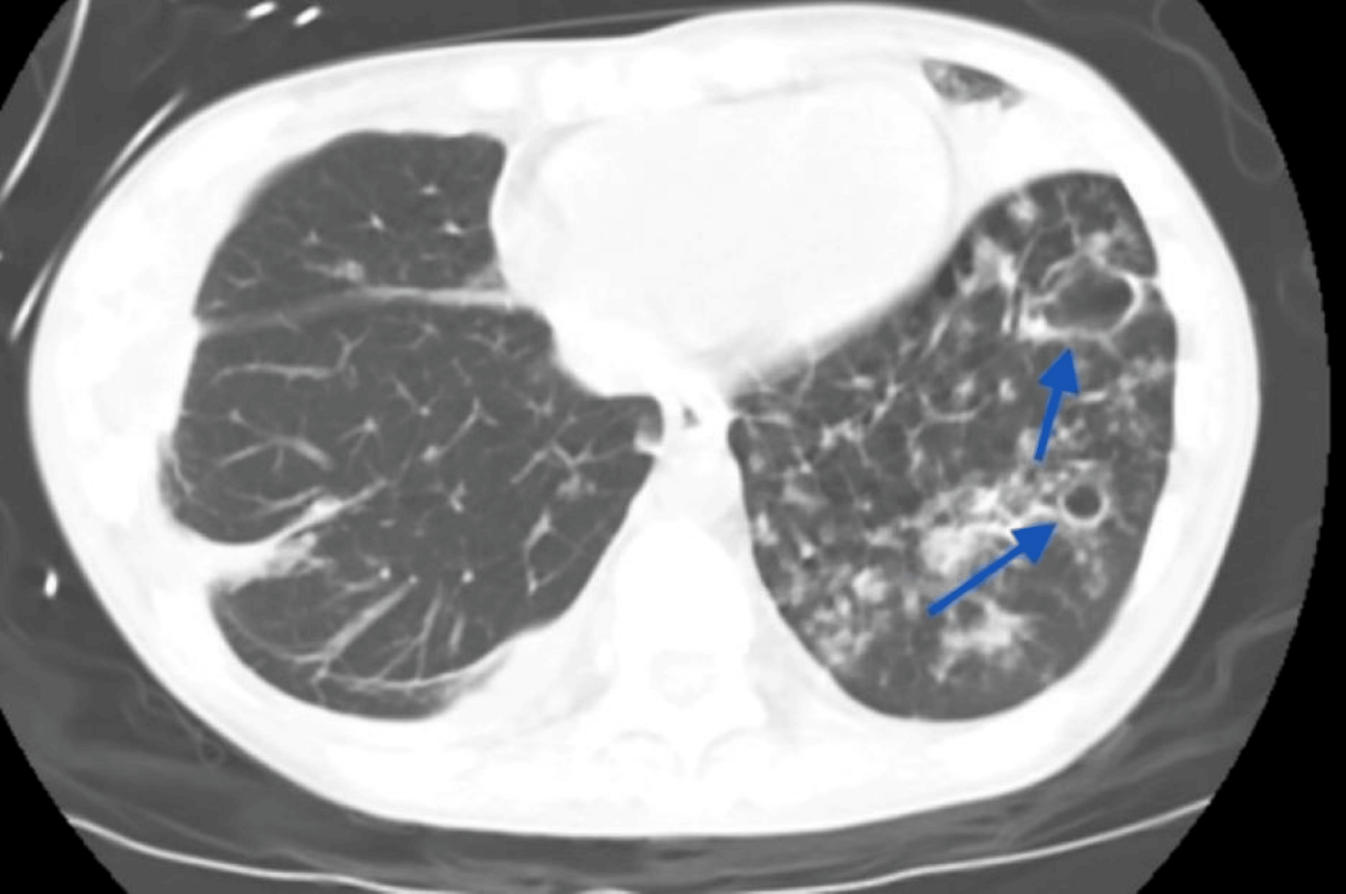
CT of the abdomen and pelvis revealing bibasilar reticulonodular opacities and left basilar cavitary lesions (blue arrows).

CT of the abdomen and pelvis with oral contrast revealed wall thickening of the cecum and ascending colon, suggestive of colitis (Figure 2), as well as bibasilar reticulonodular opacities and left basilar cavitary lesions concerning for infection/inflammation with suspicion for mycobacterial organisms (Figure 3).

**Figure 4:**
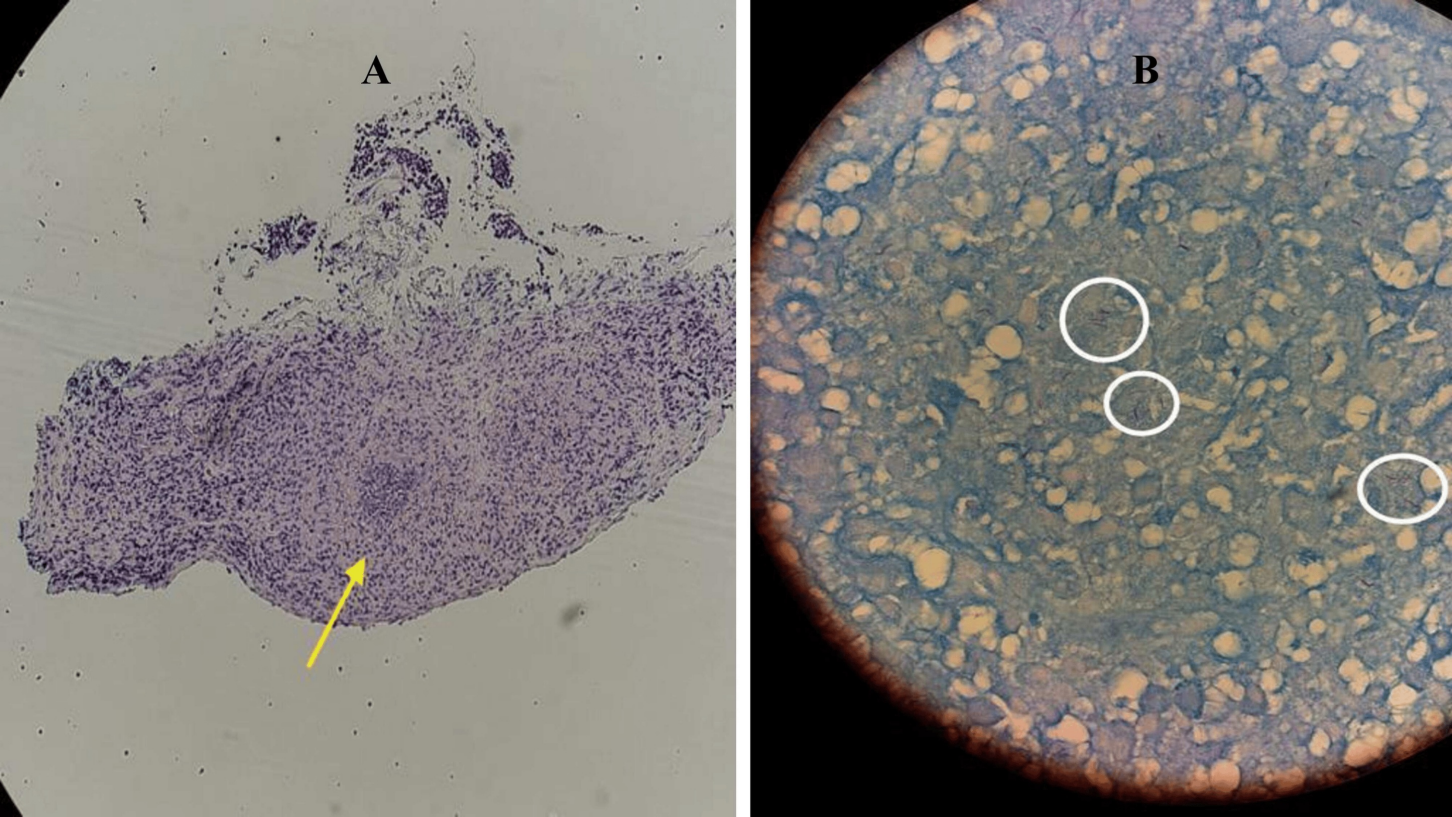
Histopathology findings showing acid granulomatous colitis and acid-fast bacilli. A: Active granulomatous colitis and granulomas (yellow arrow). B: Acid-fast bacilli (white circle) confirmed with Ziehl-Neelsen stain in the colon.

Acid-fast bacilli smear x3 results were positive for bacterial tuberculosis. Colonoscopy findings revealed a deep, irregular cecal ulcer with a white base 0.7-0.9 cm in size, multiple deep, irregular transverse colon ulcers 0.6-1 cm in size, and irregular descending colon ulcers with white bases. All ulcers were biopsied and sent for examination. Result of the biopsy revealed diffuse granulomatous colitis in the cecum (Figure 4A), and active granulomatous colitis in the ascending and descending colon. Ziehl-Neelsen stain of the specimen was positive for acid-fast bacilli (Figure 4B).

The patient was started on a course of antituberculosis medications. She was subsequently followed up in the outpatient clinic. Her symptoms and weight improved significantly with treatment.

## Discussion

High incidence of TB in the past two decades is mostly attributed to record HIV infection, elevated drug resistance, economic downturn, and immigration from endemic areas [7,8]. An estimated 8.7 million new TB cases and 1.4 million deaths were reported in 2011 [9]. The rise in abdominal TB is also consistent with these findings [8]. A study revealed that 21% of all TB cases diagnosed in the United States in 2009 involved extrapulmonary manifestations, including the presence of intra-abdominal disease [10]. Abdominal tuberculosis has been reported as the sixth most common form of extrapulmonary manifestation of tuberculosis [11]. Meanwhile, ITB only accounts for less than 1% of all documented abdominal tuberculosis cases [12]. ITB is difficult to diagnose, particularly when patients present with gastrointestinal symptoms without any form of pulmonary infection. Its ability to mimic Crohn's disease, colonic adenocarcinoma, and infectious colitis could easily result in misdiagnosis [5]. Although ITB could affect any segment of the gastrointestinal tract, ileocecal involvement is mostly seen due to the presence of rich lymphoid tissue and relative stasis of intestinal contents [13]. ITB is a rare extrapulmonary presentation of tuberculosis, and it is mostly seen in developing countries [3]. It can be diagnosed in patients presenting with a combination of persistent lung disease and gastrointestinal symptoms or as an isolated finding in patients presenting with other manifestations (abdominal pain, weight loss, diarrhea, generalized weakness, fever, etc.) [3]. Typically, inflammatory bowel disease and intestinal cancer must be ruled out in these patients during work-up because of the similarities in presentation and significant differences in treatment modalities. Hence, ITB work-up may involve the combination of radiologic, histopathological, molecular, and microbiological techniques. Early colonoscopy, tissue biopsy, AFB smear, and molecular tests such as PCR are very important during work-up. Our patient was hypoxic at presentation, hence the need to explore the possibility of a pulmonary infection. Meanwhile, she also had gastrointestinal symptoms alongside which warranted further work-up. Profound weight loss and malnutrition seen in this patient were probably due to the presence of lingering chronic TB, which was not promptly diagnosed and treated. Caseating granulomas with AFB positivity and PCR tests helped to distinguish TB colitis from Crohn’s disease. Patients diagnosed with ITB typically respond to antituberculosis medications. Treatment for ITB involves a two-phase medication regimen: an initial two months of rifampin, isoniazid, pyrazinamide, and ethambutol, followed by four to six months of rifampin and isoniazid [14]. Our patient received antituberculosis medications based on established guidelines. Subsequent outpatient follow-up after discharge showed significant improvement in her symptoms, including weight gain. This case further reinforces the need for extrapulmonary TB work-up in immunocompetent individuals presenting with atypical gastrointestinal symptoms.

## Conclusions

Tuberculosis may have unique presentations outside pulmonary manifestations. ITB remains a diagnostic challenge because it closely mimics inflammatory bowel disease, colonic adenocarcinoma, and infectious colitis. Clinicians should consider ITB as a differential diagnosis in patients presenting with diarrhea, abdominal pain, and weight loss, especially in the presence of constitutional symptoms and lung findings. The diagnosis of ITB may involve investigation with a combination of radiologic, histopathological, molecular, and microbiological techniques. Accurate and timely diagnosis of ITB could forestall risks associated with extensive diagnostic procedures and use of inappropriate medications. Antituberculosis medications remain the standard of care for the treatment of ITB.
